# Tim-3 inhibits low-density lipoprotein-induced atherogenic responses in human umbilical vein endothelial cells

**DOI:** 10.18632/oncotarget.17720

**Published:** 2017-05-09

**Authors:** Ming-Ke Qiu, Song-Cun Wang, Yong Tang, Chang Pan, Yang Wang, Shu-Qing Wang, Zhi-Wei Quan, Jing-Min Ou

**Affiliations:** ^1^ Department of General Surgery, Xinhua Hospital, Shanghai JiaoTong University, School of Medicine, Shanghai, China; ^2^ Department of Gynecology, Xinhua Hospital, Shanghai JiaoTong University, School of Medicine, Shanghai, China; ^3^ Department of Cardiology, Xinhua Hospital, Shanghai JiaoTong University, School of Medicine, Shanghai, China

**Keywords:** atherosclerosis, Tim-3, ox-LDL, inflammatory reaction, HUVEC

## Abstract

Endothelial injury and dysfunction followed by endothelial activation and inflammatory cell recruitment are factors contributing to the initiation and progression of atherosclerosis. Oxidized low-density lipoprotein (ox-LDL) promotes inflammation during atherogenesis and lipid deposition in the arterial wall. We observed that stimulation of human umbilical vein endothelial cells (HUVECs) with ox-LDL activated pro-inflammatory cytokine production and apoptosis, inhibited cell migration, and upregulated T-cell immunoglobulin and mucin domain 3 (Tim-3) expression. Tim-3, in turn, protected HUVECs from ox-LDL-induced apoptosis via the JNK pathway and reversed the inhibition of migration. Tim-3 also inhibited ox-LDL-induced inflammatory cytokine production by suppressing NF-κB activation. In addition, Tim-3 increased production of type 2 T helper cells (Th2) and regulatory T cell (Treg)-associated cytokines. Blocking Tim-3 reversed its effects on the inflammatory response to ox-LDL. Thus, Tim-3 signaling may be a “self-control” mechanism in ox-LDL-triggered inflammation in HUVECs. These results identify Tim-3 as a factor in HUVEC activity and suggest its potential in the treatment of atherosclerosis.

## INTRODUCTION

Atherosclerosis is a chronic inflammatory disease of blood vessels. Atherosclerosis lesions contain monocytes, macrophages, T cells, other immune cells, and cholesterol. Atherosclerosis was believed to be the passive accumulation of cholesterol in the vessel wall. Evidence indicates that atherosclerosis is a chronic inflammatory disease [1]. Early lesions called fatty streaks consist of sub-endothelial depositions of lipids, macrophage foam cells loaded with cholesterol, and T cells. Over time, a more complex lesion develops, with apoptotic and necrotic cells, cell debris, and cholesterol crystals forming a necrotic core in the lesion [2].

Normal blood vessels require the morphological integrity and normal function of endothelial cells. Endothelial cell injury and dysfunction lead to impaired vessel barrier function. Under these conditions, lipids and mononuclear cells are more likely to deposit on the intimal layer. Subsequently, foam cell formation and innate inflammatory responses lead to atherosclerosis. The intima, the innermost layer of the arterial wall, protects against inflammation, inhibits thrombosis, and is a factor in atherosclerosis. Endothelial cells are the major cellular component of the intima. They form anti-inflammatory signaling networks and prevent excessive reactions to pathogens.

Oxidized low-density lipoprotein (ox-LDL) is a factor in the initiation and progression of atherosclerosis, and it contributes to endothelial dysfunction and plaque destabilization through multiple mechanisms [3]. The oxidation hypothesis of atherosclerosis proposes that ox-LDL induces foam cell formation, alters nitric oxide signaling, initiates endothelial activation, and stimulates the expression of adhesion molecules that accelerate leukocyte homing to the atherosclerosis site [4]. Ox-LDL can damage endothelial cells, enhance endothelial cell adhesion, and induce pro-inflammatory factor, adhesion molecule, and chemokine expression on vascular endothelial cells and mononuclear macrophages. Ox-LDL, in combination with lectin-like oxidized low-density lipoprotein receptor 1, can produce many effects, including attraction of mononuclear cells into the intima, differentiation of mononuclear cells into macrophages, further transformation of macrophages into foam cells, and formation of fatty streaks (the earliest atherosclerotic lesions) [5]. Ox-LDL accelerates apoptosis in foam cells, stimulates the release of a variety of lysosomal enzymes, and promotes the development of atherosclerosis. Furthermore, ox-LDL-induced phosphorylation of mitogen-activated protein kinase (MAPK) and apoptosis in endothelial cells, which promotes endothelial dysfunction, are essential for the development of atherosclerosis [6, 7].

T-cell immunoglobulin and mucin domain 3 (Tim-3) is a regulatory molecule that facilitates the functions of several immune cells, including monocytes, macrophages, effector T cells, and regulatory T cells. Tim-3 interacts with its ligand Galectin-9 to activate apoptotic cell phagocytosis and induce immune cell apoptosis, which leads to termination of type 1 T helper (Th1) cell and T cytotoxic 1 (Tc1) cell responses, which in turn stimulate immune activities during infection, tumor growth, and organ transplantation [8]. Studies focusing on immune cells have shown that Tim-3 pathway signaling has anti-atherogenic effects during the development of atherosclerosis [9, 10]. However, whether Tim-3 stimulates the development of atherosclerosis by promoting the function of human umbilical vein endothelial cells (HUVECs) and how this pathway is activated are unknown.

We observed that Tim-3^+^ HUVECs increase expression of anti-atherogenic cytokines, indicating that Tim-3 is a factor in preventing or alleviating atherosclerosis. Ox-LDL stimulation of HUVECs upregulates Tim-3 expression, which protects HUVECs from ox-LDL-induced migration inhibition and reduces ox-LDL-trigged pro-inflammatory cytokine production and apoptosis by activating the JNK signaling pathway and subsequently inhibiting NF-κB. Furthermore, Tim-3 promotes type 2 T helper (Th2) cell cytokine bias and increases regulatory T (Treg) cell cytokine expression in HUVECs, whereas blockade of Tim-3 signaling by anti-Tim-3 monoclonal antibody (mAb) suppresses the protective effects of Tim-3. These combined results suggest that Tim-3 signaling represents a negative feedback mechanism utilized by HUVECs to avoid overstimulation of the inflammatory reaction induced by ox-LDL. Thus, Tim-3 signaling might be useful in preventing or alleviating atherosclerosis.

## RESULTS

### Tim-3 is correlated with anti-atherogenic cytokine production proliferation of HUVECs

**Figure 1:**
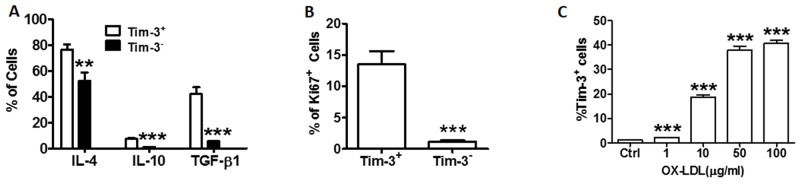
Tim-3 correlates with the production of anti-atherogenic cytokines and proliferation of HUVECs **(A)** Quantitation of flow cytometric analysis of the indicated anti-atherogenic cytokine levels (IL-4, IL-10, TGF-β) in Tim-3^+^ and Tim-3^-^ HUVECs. **(B)** Quantitation of flow cytometric analysis of Ki67+ expression in Tim-3^+^ and Tim-3^-^ HUVECs. **(C)** Quantitation of flow cytometric analysis of the proportion of Tim-3^+^ cells in HUVECs stimulated with different concentrations of ox-LDL (0, 1, 10, 50, and 100 μg/mL). Data represent mean ± SEM. **P ≤ 0.01 and ***P < 0.001 compared with the control group.

Tim-3 is an important stimulator of the immune system. To investigate whether the Tim-3 pathway is involved in the activation of HUVEC function, we examined the production of anti-inflammatory cytokines IL-4, IL-10, and TGF-β, and the expression of Ki67 in primary cultured Tim-3^+^ and Tim-3^-^ HUVECs. As shown in Figure 1A and Supplementary Figure 1, the levels of anti-inflammatory cytokines IL-4, IL-10, and TGF-β are higher in Tim-3^+^ HUVECs than in Tim-3^-^ HUVECs. Ki-67 is used as a marker of cell proliferation [11]. As shown in Figure 1B, most cells in the expanded HUVEC population were positive for Tim-3. HUVEC stimulation with different concentrations of ox-LDL upregulated Tim-3 expression in a dose-dependent correlation (Figure 1C). Levels of the Tim-3 ligand Galectin-9 also increased in ox-LDL-treated HUVECs (Supplementary Figure 2). These effects were observed when HUVECs were treated with 10 μg/mL ox-LDL. Therefore, this concentration was used for the remaining experiments.

### Tim-3 protects HUVECs from ox-LDL-induced migration inhibition

**Figure 2:**
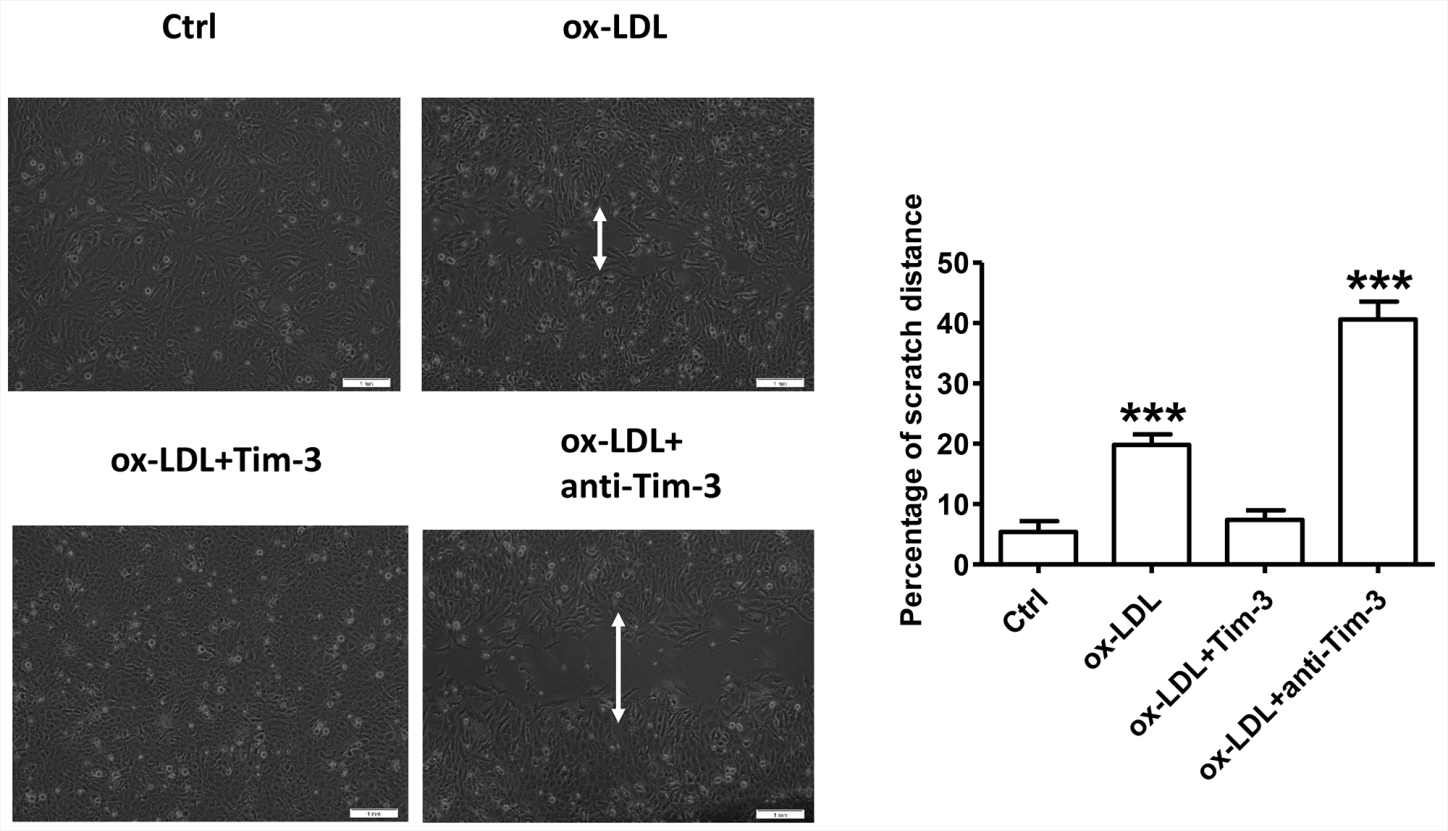
Tim-3 reverses ox-LDL-induced inhibition of HUVECs migration Wound-healing experiments were used to measure the vertical migration of HUVECs stimulated with ox-LDL (10 μg/mL) in the presence or absence of Tim-3 (1,000 ng/mL) or anti-Tim-3 mAb (10 μg/mL) after 48 hours. Representative images were obtained along the furrows after 48 hours of stimulation. The total cell numbers was counted after 48 hours of the respective treatment. The migration index was calculated by the following formula: Migration Index =Mtest/Ntest ÷ Mcon/Ncon × 100, where Mtest represents the number of migrated cells under different treatments, Ntest represents the total number of cells subjected to the respective treatments, Mcon represents the number of migrated cells under control treatment, Ncon represents the number of total cells under the corresponding control treatment. Data represent mean ± SEM. ***P < 0.001 compared with the control group.

Ox-LDL is a critical factor in endothelial dysfunction [3]. To determine the effect of ox-LDL on migration of HUVECs, these cells were subjected to the wound-healing assay as follows. HUVECs were grown to 90% confluence in culture dishes, and an open furrow was generated through the cell lawn by scratching with a pipette tip. Then, cell migration into the furrow and the restoration of cell confluency (wound healing) were documented with representative images and measured over time as the distance across the furrow in the presence of 10 μg/mL ox-LDL or vehicle control in three independent experiments. Representative images and measurements were obtained at 0, 12, 24, 36, and 48 hours after stimulation. The results showed that treatment of HUVECs with ox-LDL decelerated the restoration of cell confluency compared with that in control cells on a time-dependent basis (Supplementary Figure 3). Wound-healing experiments were also used to measure the migration of HUVECs stimulated by ox-LDL (10 μg/mL) in the presence or absence of Tim-3 (1000 ng/mL) and anti-Tim-3 (10 μg/mL) mAb after 48 hours. Tim-3 protected HUVECs from ox-LDL-induced migration inhibition, whereas administration of anti-Tim-3 mAb exacerbated the migration inhibition (Figure 2).

### Tim-3 protects HUVECs from ox-LDL-induced apoptosis by activating JNK signaling

**Figure 3:**
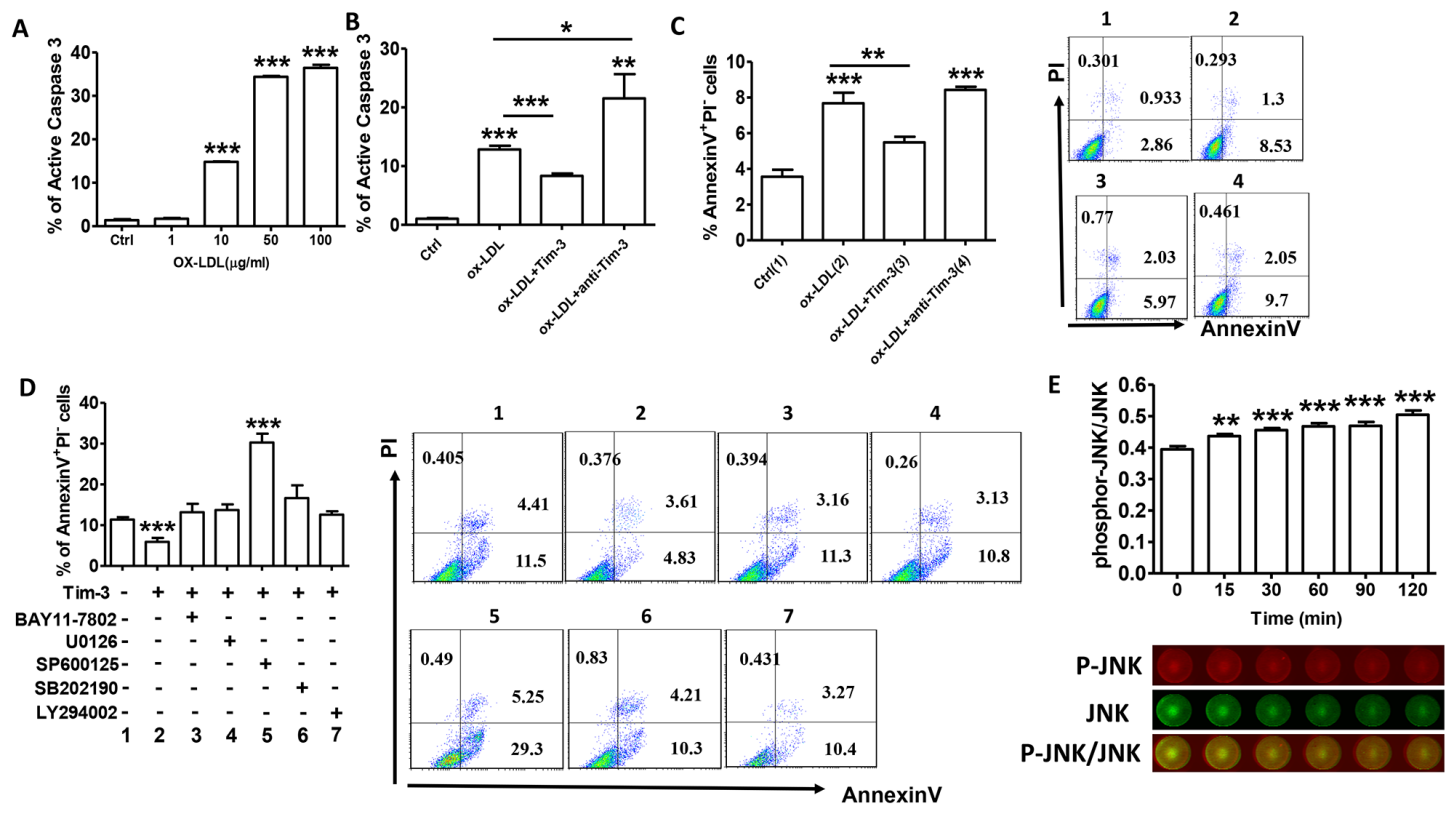
Tim-3 protects HUVECs from ox-LDL-induced apoptosis through activation of the JNK pathway **(A)** Quantitation of flow cytometric analysis of active caspase-3 expression in HUVECs stimulated with different concentrations of ox-LDL (0, 1, 10, 50, and 100 μg/mL). **(B)** Quantitation of flow cytometric analysis of apoptosis based on expression of active caspase 3 in HUVECs stimulated with ox-LDL (10 μg/mL) in the presence or absence of Tim-3 (1,000 ng/mL) or anti-Tim-3 mAb (10 μg/mL). **(C)** Flow cytometric analysis and quantitation of apoptosis based on Annexin expression and PI staining in HUVECs stimulated with ox-LDL (10 μg/mL) in the presence or absence of Tim-3 (1,000 ng/mL) or anti-Tim-3 mAb (10 μg/mL). **(D)** Flow cytometric analysis and quantitation of apoptosis in HUVECs after treatment with Tim-3 in the presence or absence of the indicated signal transduction inhibitors (NF-κB signal pathway inhibitor BAY11-7802, MAPK signal pathway inhibitor U0126, JNK signal pathway inhibitor SP600125, p38 MAPK signal pathway inhibitor SB202190, and PI3K signal pathway inhibitor LY294002). Data represent mean ± SEM. Flow cytometry plot is from one representative experiment. *P ≤ 0.05, **P ≤ 0.01, and ***P < 0.001 compared with the control group. **(E)** In-Cell Western analysis (lower) and quantitation (upper) of the JNK phosphorylation level in HUVECs treated with Tim-3 (1,000 ng/mL) for the indicated times (0, 15, 30, 60, 90, and 120 minutes). Data represent mean ± SEM. Images are representative of three independent experiments. **P ≤ 0.01 and ***P < 0.001 compared with the control group.

Knowledge of inflammatory processes has yielded new insights into the mechanisms underlying leukocyte attraction into early atherosclerosis lesions. Subsequently, increased apoptosis of endothelial cells accelerates the development of atherosclerosis [12]. Treatment of HUVECs with increasing concentrations of ox-LDL resulted in increased levels of caspase-3 (Figure 3A), indicating that ox-LDL can induce HUVEC apoptosis on a dose-dependent basis. Pretreatment with Tim-3 inhibits HUVEC apoptosis, whereas pretreatment with anti-Tim-3 mAb exacerbates apoptosis. (Figure 3B and 3C).

No signaling pathway has been precisely implicated in Tim-3 function. To further investigate the signaling pathways involved in prevention of Tim-3-induced apoptosis, the effects of specific signal transduction inhibitors on HUVEC apoptosis were examined. The results showed that most signaling pathway inhibitors, including BAY 11-7082, U1026, SB202190, and LY294002, had no effect on the inhibition of Tim-3-induced apoptosis. However, treatment with SP600125 (inhibitor of JNK signaling pathway) reversed inhibition of Tim-3-induced HUVECs apoptosis (Figure 3D). We hypothesized that Tim-3 protects HUVECs from ox-LDL-induced apoptosis through activation of the JNK pathway. To test this hypothesis, we analyzed whether treatment with Tim-3 activated JNK signaling in HUVECs. As shown in Figure 3E, administration of Tim-3 significantly increases JNK phosphorylation on a time-dependent basis. These findings suggest that Tim-3 protection of HUVECs from apoptosis might depend on the JNK signaling pathway.

### Tim-3 protects HUVECs from ox-LDL-induced proinflammatory response via NF-κB inhibition

**Figure 4:**
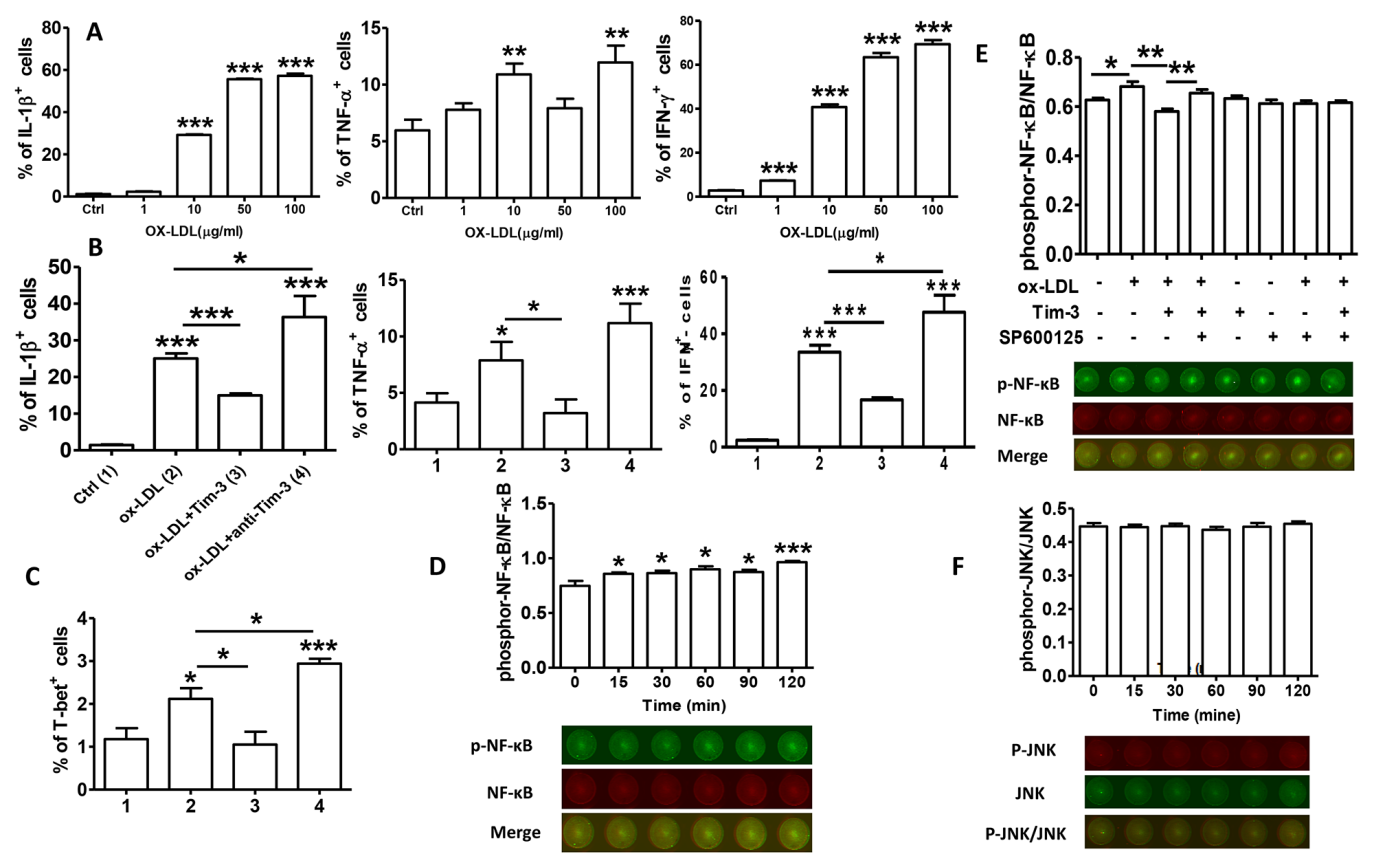
Tim-3 protects HUVECs from ox-LDL-induced proinflammatory response via NF-κB inhibition **(A)** Quantitation of flow cytometric analysis of the indicated proinflammation cytokines (IL-1β, TNF-α, and IFN-γ) in HUVECs stimulated with different concentrations of ox-LDL (0, 1, 10, 50, and 100 μg/mL). **(B and C)** Quantitation of flow cytometric analysis of the indicated proinflammation cytokines (IL-1β, TNF-α, and IFN-γ) and master transcription factor associated with Th1 cells [T-bet(C)] in HUVECs stimulated with ox-LDL (10 μg/mL) in the presence or absence of Tim-3 (1,000 ng/L) or anti-Tim-3 mAb (10 μg/mL). Data represent mean ± SEM. *P ≤ 0.05, **P ≤ 0.01, and ***P < 0.001 compared with the control group. **(D)** In-Cell Western analysis (lower) and quantitation (upper) of the level of NF-κB phosphorylation in HUVECs stimulated with ox-LDL (10 μg/mL) for the indicated times (0, 15, 30, 60, 90, and 120 minutes). **(E)** In-Cell Western analysis (lower) and quantitation (upper) of phosphorylation/activation of NF-κB in HUVECs stimulated with ox-LDL (10 μg/mL) in the presence or absence of Tim-3 and the indicated inhibitors of JNK signal transduction, alone or together. **(F)** In-Cell Western analysis (lower) and quantitation (upper) of the level of JNK phosphorylation in HUVECs stimulated with ox-LDL (10 μg/mL) for the indicated times (0, 15, 30, 60, 90, 120 minutes). Data represent mean ± SEM. Images are representative of three independent experiments. *P ≤ 0.05, **P ≤ 0.01, and ***P < 0.001 compared with the control group.

Tim-3 is a factor in inflammatory reactions. To further explore the beneficial effects of Tim-3 in atherosclerosis, we examined proinflammatory cytokine production in HUVECs treated with ox-LDL alone and with Tim-3 or anti-Tim-3 mAb. We found that ox-LDL increases the production of proinflammatory cytokines IL-1β, TNF-α, and IFN-γ on a dose-dependent basis (Figure 4A). Tim-3 inhibits ox-LDL-induced production of IL-1β, TNF-α, IFN-γ, and T-bet, whereas anti-Tim-3 mAb reverses this effect. These data clearly show that Tim-3 controls atherosclerosis by inhibiting ox-LDL-induced inflammatory responses.

We determined the signal transduction pathways involved in the Tim-3 suppression of ox-LDL-induced inflammatory reaction in HUVECs. Early work confirmed that NF-κB is correlated with the pathogenesis of atherosclerosis [13]. More recent work verified that disturbed blood flow promotes arterial inflammation by inducing NF-κB expression in endothelial cells via JNK-ATF2 signaling [14]. We first determined whether ox-LDL treatment of HUVECs stimulated NF-κB activation at different times and found that ox-LDL treatment upregulated the level of phosphor-NF-κB compared with total-NF-κB levels on a time-dependent basis (Figure 4D). Then, we examined whether Tim-3 signaling suppressed ox-LDL-induced NF-κB activation. Because the JNK pathway was involved in apoptosis inhibition by Tim-3, we analyzed whether interference with the JNK pathway also disrupted Tim-3/ox-LDL crosstalk. To explore this possibility, HUVECs were sequentially stimulated with Tim-3, ox-LDL, and SP600125, and NF-κB phosphorylation was assessed. As shown in Figure 4E, Tim-3 suppressed ox-LDL-induced NF-κB phosphorylation. However, when SP600125 was used to block the JNK pathway, Tim-3 treatment no longer had this effect. The influence of Tim-3 itself on the phosphorylation of NF-κB was also excluded because Tim-3 alone had no effect on NF-κB phosphorylation. We also examined whether ox-LDL activated JNK signaling in HUVECs. The results (Figure 4F) demonstrated that ox-LDL stimulation did not increase the level of JNK phosphorylation. These data suggest that Tim-3 inhibits ox-LDL-induced NF-κB activation via the JNK signaling pathway.

### Tim-3 reverses ox-LDL-induced reduction of anti-atherogenic cytokine levels in HUVECs

**Figure 5:**
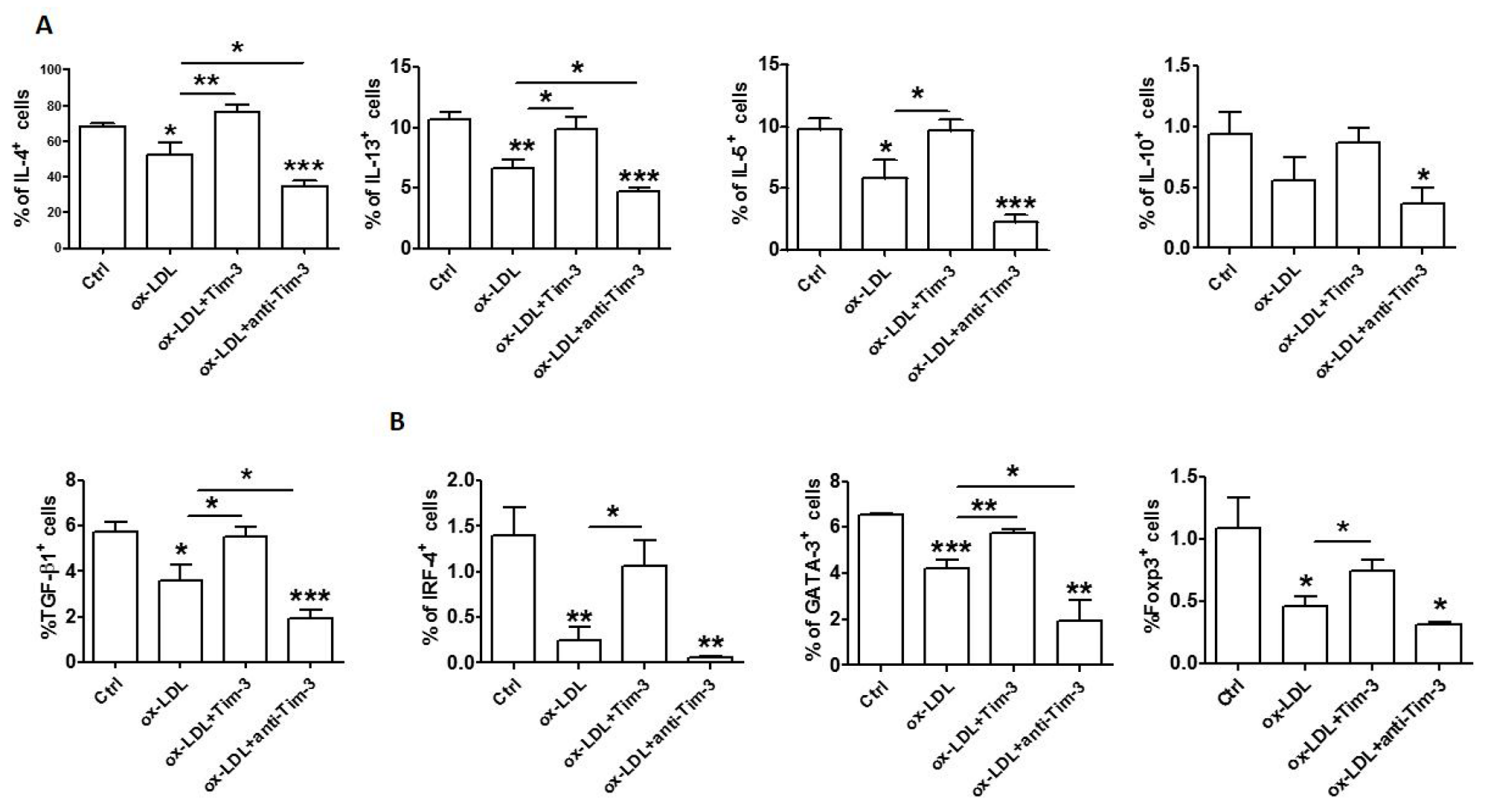
Tim-3 reverses the ox-LDL-induced decrease of anti-atherogenic cytokines in HUVECs **(A and B)** Quantitation of flow cytometric analysis of production of the indicated cytokines (IL-4, IL-13, IL-5, IL-10, and TGF-β) (A) and master transcription factors associated with Th2 and Treg cells (GATA-3, IRF-4, and FoxP3) (B) in HUVECs after stimulation with or without ox-LDL (10 μg/mL) in the presence or absence of Tim-3 (1,000 ng/mL) or anti-Tim-3 mAb (10 μg/mL). Data represent mean ± SEM. *P < 0.5, **P < 0.01, and ***P < 0.001 compared with the control group.

Tim-3 suppresses atherosclerosis. Treatment with anti-Tim-3 mAb stimulates lesion development, increases the number of monocytes/macrophages and CD4^+^ T cells, and reduces the number of regulatory T cells and regulatory B cells [9]. Given that Tim-3 suppresses ox-LDL-induced proinflammatory cytokine production and that Tim-3^+^ HUVECs produces higher levels of anti-atherogenic cytokine (IL-4, also known as Th2 cytokine; IL-10 and TGF-β, also known as Treg cytokines) than Tim-3^-^ cells, we questioned whether Tim-3 affects anti-atherogenic cytokine production in HUVECs after ox-LDL stimulation. Treatment with Tim-3 released HUVECs from ox-LDL-induced inhibition of Th2 and Treg cytokine production, which was consistent with the observation of higher anti-atherogenic cytokine expression in Tim-3^+^ HUVECs. Blocking the Tim-3 signal with anti-Tim-3 mAb reduced the production of Th2 and Treg cytokines (Figure 5A) and transcription factors (Figure 5B) in HUVECs. These data suggest that ox-LDL stimulation reduces the production of Th2 and Treg cytokines and triggers apoptosis and proinflammatory reaction in HUVECs. This finding might be another explanation for ox-LDL induction of atherosclerosis.

## DISCUSSION

In the present study, we provide the first evidence that Tim-3 correlates with higher expression levels of anti-inflammatory cytokines and greater proliferation of HUVECs. Tim-3 levels were higher when primary cultured HUVECs were stimulated with ox-LDL. Ox-LDL is a factor in the initiation and progression of atherosclerosis. Enhanced Tim-3 expression might protect HUVECs from ox-LDL-induced migration inhibition. Furthermore, Tim-3 protects HUVECs from ox-LDL-induced apoptosis through activation of the JNK pathway and proinflammatory response via NF-κB inhibition. In addition, Tim-3 reverses the decrease of anti-atherogenic cytokine levels in ox-LDL-stimulated HUVECs. Blockade of Tim-3 signaling by anti-Tim-3 mAb suppresses the protective effects of Tim-3. Combined with the results of our previous study that PD-1 and Tim-3 pathways regulate the function of CD8^+^ T cells in atherosclerosis [10], the results of this study indicates that Tim-3 forms a negative feedback loop that inhibits ox-LDL-induced inflammatory responses in HUVECs. Methods of stimulating the Tim-3 pathway provide therapeutic strategies to protect patients from ox-LDL-induced atherosclerotic lesions.

Tim-3 is a type I membrane protein. These proteins share characteristic IgV, mucin, transmembrane, and cytoplasmic domain structures [8]. Tim-3 is a factor in T cell functional differentiation and immune response. Defects in Tim family proteins lead to a variety of autoimmune diseases, infectious diseases, and tumors [15–17]. In our study, the levels of anti-inflammatory cytokines IL-4, IL-10, and TGF-β in Tim-3^+^ HUVECs were higher than those in Tim-3^-^ HUVECs, and most cells in the expanded HUVEC population were Tim-3^+^. These findings suggest that Tim-3 signals are essential to HUVECs function and inflammatory reactions. Given that Tim-3^+^ HUVECs produce more anti-inflammatory cytokines, the effect of Tim-3 on HUVECs in the differentiation of T cells should be explored in the future.

Migration of vascular endothelial cells is a factor in maintaining the integrity of blood vessel walls and contributes to the growth of new blood vessels, thereby improving the prognosis of atherosclerosis [18]. Inhibition of vascular endothelial cell migration promotes atherosclerosis progression by suppressing the repair of damaged vascular endothelial cells and angiogenesis Reendothelialization might be essential for the inhibition of smooth muscle cell proliferation and intimal hyperplasia-induced in-stent restenosis [19–20]. Tim-3 expression increased when primary cultured HUVECs were stimulated with ox-LDL. Tim-3, in turn, reduced the inhibition of HUVEC migration stimulated by ox-LDL, whereas treatment with anti-Tim-3 mAb reversed this effect. Intra-plaque angiogenesis, which depends on endothelial proliferation, is a factor in the development of atherosclerotic plaque [21]. Monolayer integrity of endothelial cells is maintained by the replacement of damaged cells via proliferation and migration of neighboring cells. Because Tim-3^+^ cells proliferated more extensively than the Tim-3^−^ cells and Tim-3 protects HUVECs from ox-LDL-induced migration inhibition, we speculate that Tim-3 promotes vascular self-repair.

We stimulated HUVECs with different concentrations of ox-LDL and found that apoptosis is induced on a dose-dependent basis. Moreover, Tim-3 pretreatment diminished ox-LDL-induced apoptosis, whereas Tim-3 blockade exacerbated this effect. When Tim-3 and anti-Tim-3 mAb were administered at the same time, their effects on ox-LDL-induced apoptosis were reciprocally neutralized. Tim-3 does not contain obvious inhibitory signaling motifs [22], and little is known about Tim-3 signaling in immune cells and HUVECs. We showed that Tim-3 activated the JNK pathway, and treatment with the JNK inhibitor SP600125 reversed Tim-3-induced apoptosis inhibition. JNK phosphorylation could activate numerous proliferation and survival-associated transcription factors [23]. Suppression of JNK activation could impair cell survival and induce cell apoptosis [24, 25]. The slight increase of apoptosis caused by other inhibitors might be the result of their cytotoxic effects. Therefore, we hypothesized that Tim-3 protects HUVECs from ox-LDL-induced apoptosis through activation of the JNK pathway. To test this hypothesis, we analyzed whether Tim-3 activated JNK signaling in HUVECs. As shown in Figure 3E, administration of Tim-3 increased JNK phosphorylation, suggesting that Tim-3 protection of HUVECs from apoptosis might depend on the JNK signaling pathway.

We found that ox-LDL increased the production of pro-inflammatory cytokines IL-1β, TNF-α, and IFN-γ in HUVECs on a dose-dependent basis. Tim-3 actively suppressed the ox-LDL-induced inflammatory response, reducing the production of IL-1β, TNF-α, and IFN-γ, and Tim-3 blockade inhibited this effect. Tim-3 suppressed the ox-LDL-induced pro-inflammatory response by inhibition of NF-κB activation. Although we observed that blockage of the JNK pathway eliminates Tim-3-induced NF-κB inhibition, the precise mechanism by which Tim-3-induced JNK activation inhibits ox-LDL signaling remains obscure. In addition, ox-LDL had no effect on JNK phosphorylation in HUVECs. We speculate that Tim-3 signaling enhances JNK activation, which inhibits ox-LDL-induced NF-κB activation, and then reduces NF-κB-driven production of pro-inflammatory cytokines by a still unknown mechanism in HUVECs.

Studies report that Th1-type cytokines are pro-atherogenic and Th2-type and Treg-type cytokines are athero-protective [26–30]. We showed that Tim-3 protected HUVECs from ox-LDL-induced apoptosis and proinflammatory responses, and ox-LDL reduced the production of Th2 and Treg cytokines. Treatment of HUVECs with Tim-3 enhanced IL-4, IL-13, IL-5, IL-10, TGF-β, GATA-3, IRF-4, and FoxP3 production, which were reduced by Tim-3 blockade. Tim-3 usually functions as a receptor, so treatment of cells with exogenous Tim-3 might inhibit Tim-3 signal transmission because of saturation binding of Tim-3 ligands (e.g., Galectin-9). Treatment with exogenous soluble Tim-3 blocked the inhibitory effect of Tim-3 [31, 32]. These previous results suggest that treatment of HUVECs with exogenous recombinant Tim-3 should augment the pro-inflammatory responses induced by ox-LDL. However, this phenomenon has also been reported in decidual stromal cells [33]. This response might be caused by the direct interaction of exogenous Tim-3 and the putative Tim-3 ligand Galectin-9, which was strongly expressed in HUVECs in the presence of ox-LDL. Tim-3 might function as a ligand for negative signaling in HUVECs, but whether soluble Tim-3 Ig mimics the function of native soluble Tim-3 remains unknown. Therefore, we conclude that treatment with exogenous soluble Tim-3 did not facilitate ox-LDL-induced apoptosis and proinflammatory reaction in HUVECs, but rather suppressed these reactions. Nonetheless, further experiments are needed to identify the mediators and factors that promote the observed results.

Our data demonstrate a key function of Tim-3 in a negative feedback loop that inhibits ox-LDL-induced apoptosis and proinflammatory reaction in HUVECs. The results also highlight the functional role of Tim-3 in non-classical immune cells. These findings identify Tim-3 as a promotor of HUVEC function, which might be critical for the development of atherosclerosis. Methods of promoting the Tim-3 pathway provide novel therapeutic strategies to inhibit the development of atherosclerotic lesions and prevent cardiovascular disease.

## MATERIALS AND METHODS

### Cell culture

HUVECs were purchased from Lonza (Walkersville, MD, USA). Cells were grown in Biorich containing 20% fetal bovine serum (FBS), 50 µg/mL endothelial cell growth supplement (BD Biosciences), and 50 µg/mL heparin (Sigma). Cells were cultured at 37°C in a humidified atmosphere with 5% CO_2_.

### Treatment of HUVECs

HUVECs were cultured overnight in complete medium, and then incubated in serum-free medium for 12 hours, followed by stimulation with a range of ox-LDL concentrations (0, 1, 50, and 100 µg/mL) for 48 hours. HUVECs were collected for flow cytometric analysis. Selected cell cultures were treated with 1,000 ng/mL recombinant Tim-3 (2365-TM-050, R&D Systems, Minneapolis, MN, USA), 10 mg/mL anti-Tim-3 mAb (clone F38-2E2, Biolegend, San Diego, CA, USA), NF-κB inhibitor BAY 11-7082 (30 µM), ERK1/2 inhibitor U0126 (30 µM), JNK signaling pathway inhibitor SP600125 (30 µM), P38 signaling pathway inhibitor SB202190 (30 µM), or PI3K signaling pathway inhibitor LY294002 (30 µM).

### Flow cytometry

Expression of cell surface molecules and intracellular cytokines was evaluated by use of flow cytometry. Cells were selectively marked with the following probes: Phycoerythrin (PE)-conjugated anti-human Tim-3 or Galectin-9; active Caspase-3, GATA-3, or T-bet (Biolegend); Fluorescein isothiocyanate (FITC)-conjugated anti-IFN-γ, IL-1β, or Foxp3 (Biolegend); PE/CY7-conjugated IL-10 or TGF-β (Biolegend); APC-conjugated IL-5, IL-13, or TNF-α (Biolegend); Brilliant Violet 421-conjugated IL-4 or Ki-67 (Biolegend); and anti-human IRF4 eFluorH 660 (eBioscience, San Diego, CA, USA). For intracellular staining, cells were fixed and permeabilized by use of the Fix/Perm Kit (Biolegend). Cell fluorescence was measured by acquiring a minimum of 10,000 events by a Beckman-Coulter CyAN ADP flow cytometer (Brea, CA, USA) and analyzed by FlowJo software (Tree Star, Ashland, OR, USA).

### Annexin V and propidium iodide (PI) staining for cell apoptosis

HUVECs were seeded at 2×10^5^ cells/well in 24-well plates, incubated overnight, and treated with ox-LDL, Tim-3, anti-Tim-3, and/or selected signal transduction inhibitors. Cells were harvested and resuspended in 100 mL annexin-binding buffer containing 5 mL FITC-annexin V and 1 mL PI working solution (BD Biosciences, Franklin Lakes, NJ, USA), and then incubated in the dark for 15 minutes at room temperature. An additional 400 mL binding buffer was added, and HUVECs were analyzed immediately by flow cytometry (BD Biosciences).

### Wound-healing experiments

HUVECs migration was measured by determination of the ability of cells to move into an acellular space. Linear furrows were scored through a lawn of HUVECs that had grown to 90% confluence. The cells were allowed to grow, and the width of the furrow (wound) was monitored at selected times after treatment under a phase-contrast microscope. Photographs were taken and the relative distance traveled by the cells at the acellular front was measured at five different marked crossing locations per well.

### In-cell western assay

In-Cell Western analysis was carried out to determine intracellular levels of NF-κB, JNK, and the phosphorylated forms of NF-κB and JNK, according to the protocol described by Egorina [34]. Primary cultures of HUVECs in 96-well plates were cultured in serum-free medium for 12 hours; stimulated with 1 mg/mL Tim-3 or 10 μg/mL ox-LDL for 0, 30, 60, 90, or 120 minutes; and followed by fixation, infiltration, and blocking. Then, cells were incubated with rabbit anti-human JNK (1:50) and mouse anti-human phospho-JNK (1:50) antibodies at 4°C overnight. Cells were incubated with the following secondary antibodies: IRDye700DXH-conjugated affinity-purified (red fluorescence) anti-mouse antibody and IRDye800DXH-conjugated affinity purified (green fluorescence) anti-rabbit antibody (both from Rockland, Gilbertsville, PA, USA). Images of target molecule fluorescence were obtained by use of the Odyssey Infrared Imaging System (LI-COR Biosciences). Expression of phosphorylated proteins was calculated as the ratio of the intensity of staining of phosphorylated protein to that of the corresponding total protein. To examine NF-κB activation level, HUVECs cultured in 96-well plates were sequentially stimulated with Tim-3, ox-LDL, and SP600125. NF-κB phosphorylation was assessed using mouse anti-human NF-κB p65 (1:50) and rabbit anti-human phospho–NF-κB p65 (Ser 311) (1:50). These experiments were carried out in triplicate and repeated three times.

### Statistical analysis

Statistical significance of differences between two groups was determined by the post-hoc Dunnett *t*-test. Multiple groups were analyzed by one-way or two-way ANOVA with Bonferroni post-tests by Prism Version 5 software (GraphPad, San Diego, CA, USA). For all statistical tests, *P* values < 0.05 were considered as statistically significant.

### SUPPLEMENTARY MATERIALS FIGURES


