# Supplementary material for: Tim-3 inhibits low-density lipoprotein-induced atherogenic responses in human umbilical vein endothelial cells
Source: Oncotarget. 2017 May 9;8(37):61001–10. doi: 10.18632/oncotarget.17720 (PMC5617401; doi:10.18632/oncotarget.17720)
Supplement: Supplementary file 1 [file oncotarget-08-61001-s001.pdf]

Tim-3 inhibits low-density lipoprotein-induced atherogenic responses in human umbilical vein endothelial cells

SUPPLEMENTARY MATERIALS

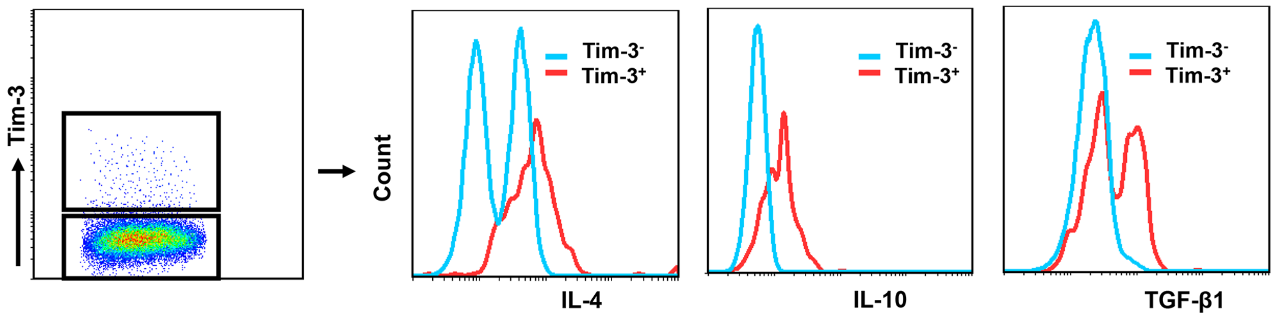

Supplementary Figure 1: Tim-3 correlates with the production of anti-atherogenic cytokines of HUVECs. Flow cytometric analysis of the indicated anti-atherogenic cytokine levels (IL-4, IL-10, and TGF-β) in Tim-3^{+} and Tim-3^{-} HUVECs.

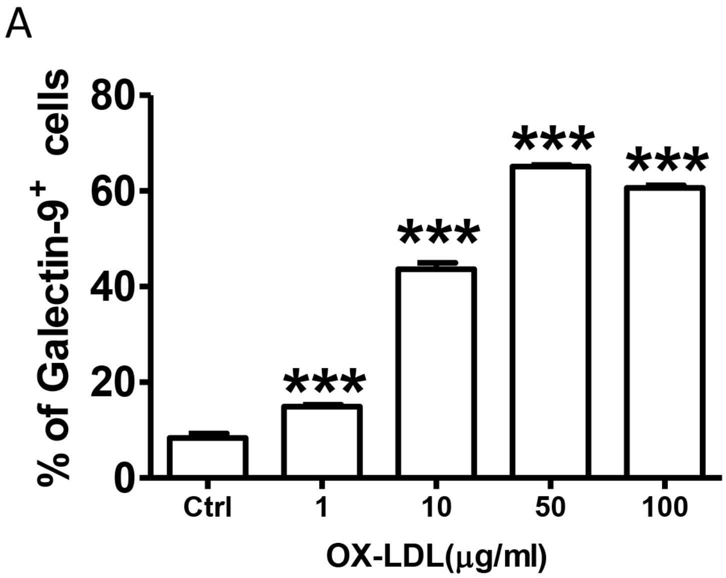

Supplementary Figure 2: Ox-LDL up-regulates the expression of Galectin-9 in HUVECs. Quantitation of flow cytometric analysis of proportion of Galectin-9+ cells in HUVECs stimulated with different concentrations of ox-LDL (0, 1, 10, 50 and 100 μg/mL). Data represent mean ± SEM. ***$P < 0.001$ compared with the control group.

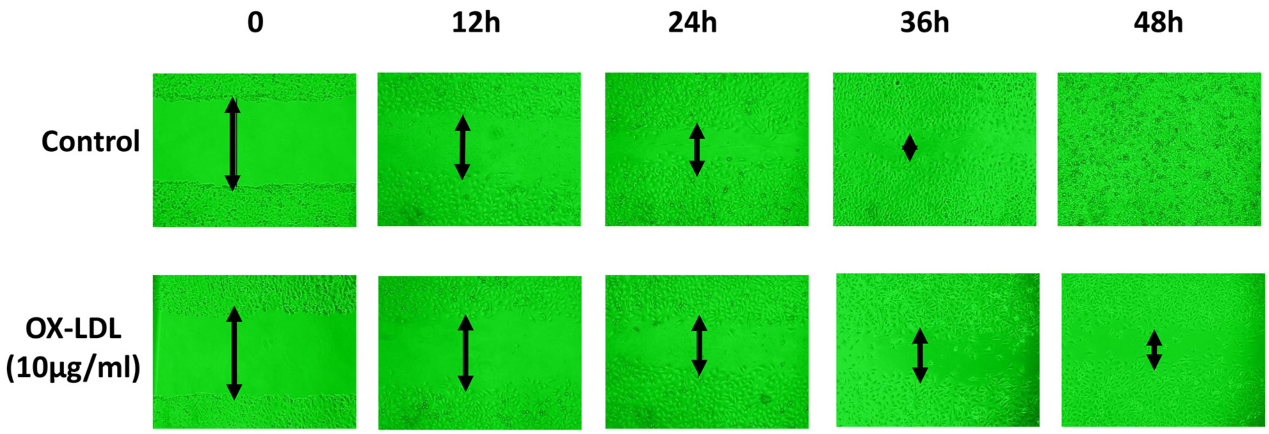

Supplementary Figure 3: Ox-LDL slows down the restoration of cell confluency by HUVECs. Wound-healing assay in HUVECs treated with ox-LDL (10 µg/mL). Distance between the furrow edges in the control (upper) or ox-LDL-treated (lower) cells in three independent experiments was measured and presented graphically as percentage of the initial distance (0 hours). Representative images were obtained along the furrows at 0, 12, 24, 36, and 48 hours after stimulation with 10 µg/mL ox-LDL or vehicle.
